# A Systematic Review of the (Un)known Host Immune Response Biomarkers for Predicting Recurrence of Urinary Tract Infection

**DOI:** 10.3389/fmed.2022.931717

**Published:** 2022-07-04

**Authors:** Iva Sorić Hosman, Andrea Cvitković Roić, Lovro Lamot

**Affiliations:** ^1^Department of Pediatrics, Zadar General Hospital, Zadar, Croatia; ^2^Department of Nephrology and Urology, Clinic for Pediatric Medicine Helena, Zagreb, Croatia; ^3^School of Medicine, Josip Juraj Strossmayer University of Osijek, Osijek, Croatia; ^4^Division of Nephrology, Dialysis and Transplantation, Department of Pediatrics, University Hospital Centre Zagreb, Zagreb, Croatia; ^5^Department of Pediatrics, University of Zagreb School of Medicine, Zagreb, Croatia

**Keywords:** recurrent urinary tract infections (rUTI), biomarkers, urinary tract innate immunity, urothelium, uroepithelial cell, antimicrobial peptides (AMPs), vitamin D deficiency

## Abstract

Recurrent urinary tract infections (rUTI) represent a major healthcare and economic burden along with a significant impact on patient’s morbidity and quality of life, even in the absence of well-known risk factors, such as vesicoureteral reflux. Despite numerous attempts to find a suitable therapeutic option, there is no clear benefit of any currently available intervention for prevention of UTI recurrence and its long-term consequences such as hypertension, renal scarring and/or insufficiency. The common treatment practice in many centers around the globe involves the use of continuous low-dose antibiotic prophylaxis, irrespective of various studies indicating increased microbial resistance against the prophylactic drug, leading to prolonged duration and escalating the cost of UTI treatment. Moreover, the rapid appearance of multi-drug resistant uropathogens is threatening to transform UTI to untreatable disease, while impaired host-microbiota homeostasis induced by a long-term use of antibiotics predisposes patients for various autoimmune and infectious diseases. New biomarkers of the increased risk of UTI recurrence could therefore assist in avoiding such outcomes by revealing more specific patient population which could benefit from additional interventions. In this light, the recent findings suggesting a crucial role of urothelial innate immunity mechanisms in protection of urinary tract from invading uropathogens might offer new diagnostic, prognostic and even therapeutic opportunities. Uroepithelial cells detect uropathogens *via* pattern recognition receptors, resulting in activation of intracellular signaling cascade and transcription factors, which ultimately leads to an increased production and secretion of chemokines, cytokines and antimicrobial peptides into the urinary stream. Emerging evidence suggest that the disturbance of a single component of the urinary tract innate immunity system might increase susceptibility for rUTI. The aim of the current review is to update clinicians and researchers on potential biomarkers of host immune response alterations predisposing for rUTI and propose those well worth exploring further. For this purpose, over a hundred original papers were identified through an extensive PubMed and Scopus databases search. This comprehensive review might enrich the current clinical practice and fill the unmet clinical needs, but also encourage the development of therapeutic agents that would facilitate urinary bacterial clearance by enhancing the host immune response.

## Introduction

Recurrent urinary tract infections (rUTI) are defined as ≥ 2 UTI in the past 6 months or ≥ 3 UTI within the preceding year (1). RUTI occur in a high percentage of patients following an initial UTI. Within one year after the initial UTI, 25–30% of adults (2, 3) and 15–30% of children (4, 5) experience UTI recurrence, even in those without any radiological or nuclear scan urinary tract abnormalities (6, 7). Children who suffer from rUTI are at increased risk of renal scarring (8) which, in the long-term, can lead to a progressive renal disease, hypertension and/or renal insufficiency in adulthood (9, 10). Consequently, rUTI represent a great health care and economic burden due to sick leave, doctor visits. antibiotic prescriptions and hospitalization expenses (3, 11, 12).

For decades, UTI recurrence was considered an omen of underlying anatomic and/or physiologic abnormalities, with renal and bladder ultrasound and voiding cystourethrography (VCUG) performed almost routinely, despite of high cost, exposure to radiation, the risk of causing UTI and/or discomfort for the patient. Though urinary tract abnormalities have been implicated as one of the most important factors predisposing to rUTI, it has become apparent that less than 40% of children with rUTI have vesicoureteral reflux (VUR) (4, 7), whereas only around 10% of adults with rUTI have any kind of urinary tract abnormalities on imaging findings (13, 14). Besides, only high grades VUR (grades 4 and 5) have been considered a risk factor for developing rUTI, while VUR grades 1 to 3 did not seem to increase the risk of developing UTI recurrence (4, 5, 15). However, some of the most recent guidelines still propose imaging studies of the urinary tract as a part of the diagnostic evaluation of both pediatric and adult patients suffering from rUTI (14, 16–20).

In patients with an UTI and a negative radiologic evaluation, recurrence is more common in females due to their anatomy (shorter urethra and a short distance from the urethral opening to the anus) and hormonal changes in pregnancy and postmenopause (2, 3). Boys who are uncircumcised are at an increased risk of infection during the first year of life (4, 6). Circumcision is associated with a 10-fold reduction in the incidence of having a UTI during the first year of life (21). In boys, recurrence is rarely seen after 2 years of age (22). In children aged 3–6 years, dysfunctional elimination syndrome is associated with a higher rUTI incidence (6, 23, 24). Additional risk factors for rUTI in adults include a childhood or family history of UTI, overrepresentation of the blood-group antigen non-secretor phenotype and P1 phenotype, behavioral factors such as increased frequency of sexual intercourse, taking on new sexual partners and use of spermicides or diaphragm for contraception and voiding disturbances that result in increased postvoiding residual urine volume (2, 25–27). The latter may be caused by neurogenic bladder dysfunction or urinary obstruction (urinary tract calculi, urethral strictures, ureteropelvic junction obstruction, ureterocele, malignancies, pelvic prolapse in women or benign prostatic hyperplasia in men) (14, 28).

Continuous antibiotic prophylaxis (CAP) has become standard of care for prevention of rUTI and long-term renal damage in both children and adults suffering from rUTI. However, multiple recently conducted meta-analyses based on a large number of randomized controlled trialshave shown CAP has no or a minimal effect in reducing the recurrence of UTI and/or renal scarring in children (15, 29–31). Nevertheless, despite these recent findings, many professional societies still recommend administration of CAP in all patients with rUTI (1, 16–20). Moreover, depending on imaging findings, some patients might even be submitted to operative procedures. Unfortunately, regardless of all medical interventions, UTI still reoccurs in a substantial number of patients, suggesting other factors influencing rUTI susceptibility (32).

Beside the lack of efficacy in the prevention of rUTI, long-term low-dose antibiotics does not seem to reduce the appearance and progression of permanent renal damage, while increasing the risk of microbial resistance against the prophylactic drug in breakthrough infections (33). Consequently, a high proportion (up to 50%) of patients with rUTI need to modify antibiotic treatment (11). Moreover, an accumulating wealth of evidence have shown that antibiotics might have detrimental effect on host-microbiota homeostasis, posing a serious menace to the global public health (34, 35). These imbalances in gut microbiota, or dysbiosis induced by early use of antibiotics, have been linked to obesity, allergy and atopic disorders, autoimmune diseases such as type I diabetes, rheumatoid arthritis and multiple sclerosis, along with various infectious diseases (35, 36). Therefore, the possible effect of antibiotics on immune function needs to be accounted when developing effective prophylactic and therapeutic strategies in infants and children, but also in adults.

With rapidly growing problem of antibiotic overuse on one hand and severe consequences of rUTI for both individual and public health on other, it is becoming increasingly important to scrutinize preventive strategies for the management of rUTI. One of the approaches is a discovery of a host biomarker of rUTI susceptibility that could predict future UTI recurrence after the first UTI. Finding this multipotent predictor might allow targeted interventions, such as various non-surgical endoscopic and surgical procedures or continuous antibiotic prophylaxis, only in patients with a clear benefit. Moreover, it could lead to the rationalization of both diagnostic and therapeutic interventions and, consequently, reduced trauma to patients, especially children, as well as immense health care savings.

Over the years, a large body of knowledge has accumulated about factors influencing the individual susceptibility to rUTI, from gene to protein level. It has been suggested that UTI is a product of a complicated host-bacteria interaction, while susceptibility to rUTI is a consequence of host defense deficiencies (37). Innate immunity seems to play a key role in maintaining homeostasis and shielding urinary tract from invading uropathogens. When these innate defense mechanisms are defective, pathogen susceptibility increases, resulting in rUTI. Significant efforts have been also made to translate newly discovered innate urinary tract defense mechanisms into clinically efficient markers predictive of rUTI as well as therapeutic targets enhancing urinary tract innate immunity. Therefore, in the presented systematic review we aimed to summarize various studies examining the host defense biomarkers with a potential predictive value for rUTI.

## Search Methods

**FIGURE 1:**
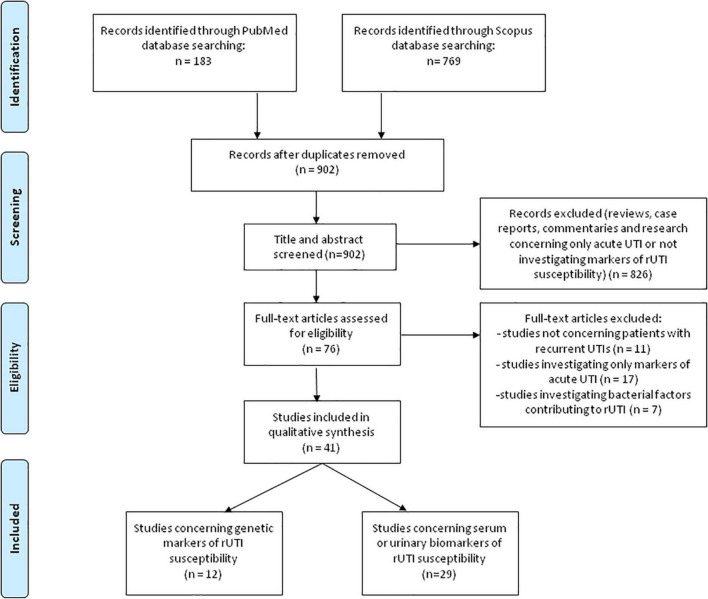
PRISMA-flowchart (38) illustrating the literature search and study selection process.

A comprehensive literature review was performed using PubMed and Scopus databases to identify articles exploring the potential genetic or protein markers of host defense deficiency in patients susceptible for rUTI, according to the published guidance on systematic reviews (38). We used search term of “recurrent urinary tract infection” in combination with “marker” or “biomarker.” Search terms were used in all fields including keywords, MeSH terms or any text word to maximize the output from the literature. Only available full-text articles in English published until December 1*^st^* 2021 were included. Additional exclusion criteria were case reports, reviews, commentaries and studies not concerning patients with rUTI or not discussing potential genetic predictors or biomarkers of host defense deficiency leading to rUTI susceptibility. Reference lists of the selected articles were reviewed to identify additional articles meeting the eligibility criteria. The database search resulted in 952 articles of which 76 remained after the removal of duplicates and title/abstract screening. Finally, after assessing the full-text articles for eligibility and screening of the reference lists, a total of 41 full-text articles were included and thoroughly analyzed in the present review (Figure 1 – PRISMA flowchart). All studies in which genetic polymorphisms or urine or serum biomarkers were accessed for predicting rUTI were included in this review. The results of human studies concerning genetic susceptibility for rUTI (*n* = 12) are summarized in Table 1, whereas human studies concerning urine or serum biomarkers of increased risk for rUTI (*n* = 29) are summarized in Table 2.

**TABLE 1:** Characteristics and conclusions of studies investigating genetic polymorphisms as biomarkers for predicting recurrent urinary tract infections (rUTI).

Study (reference number)	Investigated genes	Number of subjects (characteristics): -subgroups	Study conclusions
Hawn et al. (41)	*TLR1, TLR2, TLR4, TLR5*	1261 (women, 18–49 years): -431 with rUTI history -400 acute UTI -430 healthy controls	*TLR2 G2258A* and *TLR5 C1174T* are associated with higher risk for developing rUTI, whereas *TLR4 A896G* and *TLR1 G1805T* seem to have a protective role in rUTI.
Karoly et al. (42)	*TLR4* and *HSPA1B*	338 (children, 2.8–12 years): -103 with rUTI history, but without VUR -235 healthy controls	*TLR4 896G* and *HSPA1B 1267G* allele carriers have increased risk for developing rUTI, independently of VUR. HSPA1B 1267GG genotype is a risk factor for renal scarring, independent of VUR.
Tabel et al. (45)	*TLR2*	240 (children and unrelated adults): -124 (children, 0–18 years): 24 with a single UTI and 100 with rUTI -116 healthy controls (unrelated adults)	*TLR2 Arg753Gln* allele frequency is significantly higher in patients with rUTI than in those with a single UTI and could be a risk factor for developing rUTI.
Fischer et al. (48)	*IRF3 promoter*	198 (children and unrelated adults): -64 children (21 with rUTI, 16 with asymptomatic bacteriuria, 27 healthy controls) -134 adults (72 with a rUTI history and 62 healthy blood donors)	*IRF3 promoter* efficiency is reduced by the SNPs at the -925 and -776 positions which occur in about 80% of rUTI patients compared to less than 20% of healthy controls. These SNPs reduce IRF3 expression and increase the risk for rUTI.
Puthia et al. (49)	*IRF7 promoter*	149 (children and unrelated adults): -31 children (17 with rUTI, 14 with ABU) -118 adults (43 with ABU, 34 with a history of childhood rUTI and 41 healthy controls)	*IRF7* promoter SNPs with lower IRF7 expression (*rs3758650-T and rs10902179-G*) are protective against rUTI, while *IRF7* promoter SNPs with higher IRF7 expression (*rs3758650-C* and *rs10902179-A*) are a risk factor for developing rUTI.
Lundstedt et al. (65)	*CXCR1*	286 (children and adults): - 50 children, 1–12 years (7 with single UTI, 17 with rUTI, 26 age-matched controls) -236 adults (36 with rUTI, 200 healthy controls)	Single base changes in *CXCR1* resulting in decreased CXCR1 expression are associated with susceptibility to rUTI in both children and adults and are a risk factor for rUTI independent of VUR.
Javor et al. (67)	*IL-8, CXCR1 and CXCR2*	362 (children and unrelated adults): -85 children with single UTI, -62 children with rUTI -215 healthy controls (aged 3–55 years)	*CXCR1* + *2608 C* allele is associated with a higher risk of rUTI, while the *CXCR2* + *1208 T* allele has a protective role. The allele A of the *IL-8 -251 T/A* increases the risk of developing rUTI after the initial UTI.
Han et al. (68)	*CXCR1* SNPs *rs2234671* and *rs3138060*	Meta-analysis of the studies concerning the two SNPs published until December 2017	Children carrying *CXCR1* SNP *rs2234671* have a significantly increased risk of developing rUTI.
Schwaderer et al. (77)	*DEFA1A3*	593 girls from the RIVUR study: -298 with rUTI (aged < 6 years) -295 healthy controls (aged < 18 years)	Low *DEFA1A3* copy number (< 5) is a risk factor for a breakthrough rUTI independent of VUR grade.
Liu et al. (86)	*SP-A1, SP-A2, SP-D*	32 women (aged 18–70 years) with history of rUTI (without VUR or any other urinary tract abnormalities) and 30 age-matched healthy female controls	*19Ala* allele of *SP-A1* and *223Gln* allele of *SP-A2* gene lead to increased rUTI susceptibility due to low urinary SP-A expression.
Aslan et al. (110)	*VDR*	197 children: -92 with UTI, without VUR -105 healthy controls	The risk of UTI is 3.94 times higher in children carrying *Fok*I polymorphism ff genotype compared with *FF* genotype. *Apa1* polymorphism a allele (*Aa* or *aa* genotype) is a protective factor.
Mahyar et al. (111)	*VDR*	120 children: -60 with UTI, without VUR -60 healthy controls	*VDR* gene *Apa1* a allele and *Bsm1* b allele are associated with increased risk of UTI, while A and B alleles seem to be protective factors.

**TABLE 2:** Characteristics and conclusions of studies investigating serum or urine biomarkers for predicting recurrent urinary tract infections (rUTI).

Study (reference number)	Number of subjects (characteristics): -subgroups	Investigated biomarkers (measuring method)	Results
Yin et al. (44)	377 (adults, aged 19–80 years): -38 with rUTI -91 with an acute UTI -248 healthy controls	TLR4 protein expression on monocytes (flow cytometry)	Patients with rUTI have significantly lower TLR4 expression compared to those with a single UTI.
Forster et al. (51)	30 (children, aged 6 moths-21 year): -15 with rUTI history but without an active UTI -15 healthy controls	Urinary NGAL (western blot)	Urinary NGAL is significantly decreased in patients with rUTI compared to healthy controls. Local uNGAL deficiency may be a risk factor for developing UTI recurrence.
Forster et al. (54)	37 (girls, less than 4 years of age): - 24 with a single UTI -10 with rUTI, -3 controls with signs and symptoms of UTI but negative urine culture	Urinary NGAL (ELISA)	Urinary NGAL levels are not significantly different between patients with rUTI compared to those with a single UTI or those with UTI symptoms but sterile urine cultures.
Lorenzo-Gomez et al. (57)	200 elderly (aged > 65 years): -100 with rUTI history -100 healthy controls	Urinary NGAL, TGF1ß, NAG (ELISA)	Urinary NGAL, TGF1ß and NAG levels are increased in elderly with rUTI history compared to healthy controls even in between acute UTIs, possible due to renal scarring.
Yamanouchi et al. (58)	58 (children, < 4 years): -23 with a single UTI -15 with rUTI -20 healthy controls	Urinary NGAL (ELISA)	Urinary NGAL is significantly decreased in patients with rUTI compared to those with a single UTI or healthy controls. Urinary NGAL may be used as a predictor of UTI recurrence.
Frendeus et al. (63)	24 (children, aged 2–12 years): -12 with rUTI history, but no active UTI (7 with VUR, 5 without) -12 age-matched controls (admitted to hospital due to elective surgery)	Neutrophil CXCR1 expression (confocal microscopy and flow cytometry)	Children with rUTI have a reduced expression of CXCR1 that is stable over time. Reduced neutrophil CXCR1 expression is a risk factor for developing rUTI independent of VUR.
Lundstedt et al. (64)	24 (children, 1–12 years): -8 with rUTI history -2 with a single UTI in history -14 pediatric healthy controls 95 adults: - 49 adult relatives of children with rUTI -46 adult healthy controls	Neutrophil CXCR1 surface expression (flow cytometry)	Children with rUTI and their relatives have a reduced expression of CXCR1 compared to controls. Low CXCR1 expression is inherited and is one of the factors predisposing to rUTI, independent of VUR.
Smithson et al. (66)	50 premenopausal women: -20 with rUTI history without VUR or other urinary tract abnormalities (aged 21–39 years) -30 health controls (aged 18–46 years)	Neutrophil CXCR1 and CXCR2 surface expression (flow cytometry)	Patients with rUTI have significantly lower CXCR2 expression compared to healthy controls. Low CXCR2 expression on neutrophils is a risk factor for rUTI, independent of VUR.
Hannan et al. (70)	86 (women, aged 18–49 years): -41 with rUTI -45 with a single UTI	Serum cytokines and growth factors	Serum M-CSF levels are significantly higher at first UTI in women subsequently developing UTI reccurence than in those without reccurence within 3 months from the initial UTI.
Ebrahimzadeh et al. (72)	92 (Postmenopausal women, aged 63–81 years): -31 women with acute UTI and a rUTI history -35 women with rUTI but without an active infection -26 healthy controls	Urinary PGE2 (ELISA)	Elevated urinary PGE2 in an acute UTI is a predictor of UTI recurrence in postmenopausal women. Patients with the above median PGE2 concentration (> 2,318 pg/ml) during an acute UTI have a 3.5 times higher risk of UTI relapse.
Chuang et al. (75)	57 female adults: -9 with UTI recurrence during 1 year follow-up after the initial acute UTI -28 without rUTI in 1 year follow-up -20 women with urinary stress incontinence as controls	Urinary NGF (ELISA)	Urinary NGF level is significantly lower in women with UTI recurrence within 1 year after the initial UTI than in those without UTI recurrence. Urinary NGF could serve as a predictor of UTI recurrence.
Lose et al. (81)	30 girls (aged 5–17 years): -16 with rUTI history, but without urinary tract structural abnormalities -14 age-matched healthy controls	Urinary THP (radial immunodiffusion)	There are no significant differences in urinary THP expression between girls with rUTI and healthy controls.
Reinhart et al. (82)	35 women (mean age 24): -17 with rUTI history (no urinary tract structural abnormalities) -18 healthy controls	Urinary THP (ELISA)	Urinary THP concentration is not significantly decreased in women with rUTI compared with controls. THP excretion varies widely in repeat samples obtained from the same individual.
Reinhart et al. (83)	51 girls (mean age 7.9): -35 with rUTI history (no urinary tract structural abnormalities) -16 healthy controls	Urinary THP (ELISA)	Urinary THP concentration is not significantly decreased in children with rUTI compared with controls.
Liu et al. (86)	62 women (aged 18–70 years): -32 with a history of rUTI (without VUR or any other urinary tract abnormalities) -30 healthy controls	Serum and urinary SP-A and SP-D levels (ELISA)	Patients with rUTI have significantly higher serum and significantly lower urinary SP-A levels than healthy controls. Low urinary SP-A levels could be used as rUTI predictor, independent of VUR.
Suman et al. (87)	60 women (aged 20–42 years): -30 with rUTI history -30 healthy female controls	Serum and urinary antibodies to mixed coliform antigen (ELISA)	Women with rUTI have a significantly higher level of serum IgG, IgM and IgA and urinary IgG antibodies compared to controls.
Riedasch et al. (90)	74 women: -22 women with rUTI history (13 with an acute UTI, 9 with an active infection) 6 women with first acute UTI -52 adults as healthy controls	Urinary secretory IgA (ELISA)	Women with rUTI history have lower urinary sIgA than healthy controls, even during an acute UTI. Low urinary sIgA may represent one factor predisposing to recurrent UTI.
Fliedner et al. (91)	224 children: -30 girls (1–16years) with a rUTI history, but no acute infection and no VUR -11 girls (3–14 years) with acute UTI, no VUR -8 girls (4–17 years) with rUTI and VUR -175 healthy controls (94 boys, 81 girls, aged 4 days to 15 years)	Urinary secretory IgA (ELISA)	Children with rUTI have significantly lower urinary sIgA than healthy controls, while those with rUTI and an acute infection or VUR show significantly higher sIgA than healthy controls.
Floege et al. (93)	95 premenopausal women (19–49 years): -18 with rUTI history (10 without and 8 with urological abnormalities) -4 with an acute UTI and VUR -5 with selective IgA deficiency -68 healthy controls	Serum and urinary secretory IgA (ELISA)	UTI recurrence in premenopausal women is not associated with disturbances of the urinary sIgA excretion, including even a complete failure of the sIgA system.
Nielsen et al. (97)	103 premenopausal adult women: -50 with UTI -53 healthy controls	Urinary cathelicidin (ELISA)	Urine cathelicidin baseline levels of controls are significantly higher than the postinfection levels in UTI patients. Reduced cathelicidin production in the urinary tract system can raise the probability of UTI
Övünç Hacıhamdioğlu et al. (100)	74 children (aged 3 months–13 years): -36 children with UTI, without VUR -38 healthy controls	Serum 25(OH) vitamin D (ELISA) Urinary cathelicidin (ELISA)	Vitamin D deficiency in children is an independent risk factor for UTI. Urinary cathelicidin levels are significantly upregulated in children with UTI and sufficient vitamin D status, whereas urine cathelicidin levels do not increase significantly during a UTI in children with vitamin D insufficiency.
Tekin et al. (101)	146 children (aged 2 months to 6 years): -82 with UTI, without VUR -64 healthy controls	Serum 25(OH) vitamin D (chemiluminescence immunoassay)	Vitamin D deficiency in children is independently associated with UTI.
Shalabay et al. (102)	100 children (aged 2 months to 6 years): -50 with UTI, without VUR -50 healthy controls	Serum 25(OH) vitamin D (enzyme immunoassay)	Vitamin D deficiency in children is an independent risk factor for UTI.
Sadeghzadeh et al. (103)	80 children (aged 1–12 years): -40 with UTI -40 healthy controls	Serum 25(OH) vitamin D (ELISA)	Vitamin D deficiency has a significant relationship with the prevalence of UTI in children.
Mahmoudzadeh et al. (104)	150 children (aged 2–7 years): -75 with UTI -75 healthy controls	Serum 25(OH) vitamin D (chemiluminescence immunoassay)	Children with low vitamin D levels are at a greater risk of UTI compared to those with sufficient vitamin D levels.
Yang et al. (105)	238 infants (aged 1–12 months): -132 with acute UTI -106 healthy controls	Serum 25(OH) vitamin D (radioimmunoassay)	Vitamin D deficiency in infants is associated with increased risk for developing UTI. Vitamin D supplementation is associated with a decreased likelihood of UTI.
Nseir et al. (107)	186 premenopausal women (aged 20–52 years): -93 with a rUTI history -93 age-matched healthy women	Serum 25(OH) vitamin D (enzyme immunoassay)	Vitamin D deficiency in premenopausal women is an independent risk factor for rUTI.
Georgieva et al. (108)	120 children (< 3 years): - 76 with UTI (21 of them developed rUTI, VUR not excluded) -44 children with congenital hydronephrosis but without UTI	Serum 25(OH) vitamin D (chemiluminescence immunoassay) Serum cathelicidin and β-defensin-2 (ELISA)	Vitamin D deficiency is a risk factor for UTI, but is not correlated with the recurrence of infection at one-year follow-up. Vitamin D level is positively correlated with serum cathelicidin, but not with serum β-defensin-2 levels.
Muntean et al. (109)	101 children (aged 1 month to 12 years) on continuous vitamin D prophylaxis until the age of 2: -59 with UTI (42 of them with rUTI, VUR not excluded) -42 healthy controls	Serum 25(OH) vitamin D (chemiluminescence immunoassay)	Vitamin D levels are inversely associated with the risk of UTI. Vitamin D plays an important role in the prevention of UTI recurrence, as proved by lower serum levels in rUTI patients than in the ones with a first-time UTI. Vitamin D status in children with rUTI is not additionally influenced by congenital abnormalities of the urinary tract.

## Results

### Toll-Like Receptors

Toll-like receptors (TLRs) are a family of membrane receptors that recognize conserved pathogen-associated molecular patterns and initiate the innate immune response. Beside immune cells, TLRs are also expressed on non-immune cells, including epithelial cells of the urogenital tract where the initial recognition of bacteria occurs (39). Since TLRs have been acknowledged as an essential factor for bacterial activation of immune response in the urinary tract (40), they have been studied as potential predictors of UTI recurrence from gene to protein level. It has been hypothesized that specific TLR gene polymorphisms may lead to a pathogen recognition deficiency in the urinary tract and consequently may be associated with UTI recurrence (41–45). Indeed, Hawn et al. (41) found that polymorphisms of *TLR2 G2258A* and *TLR5 C1174T*, variants related with decreased signaling, are associated with an increased risk of rUTI in adult women. Contrarily, *TLR* polymorphism including *TLR4 A896G* and *TLR1 G1805T* seem to have a protective role and decreased risk of rUTI (41). Although the study involved a large cohort of middle-aged women (431 women with rUTI history, 400 women with pyelonephritis and 430 healthy controls), the subjects were not assessed for other UTI predisposing factors including VUR. Those limitations as well as age difference between studied cohorts might be the reason for opposite results obtained by Karoly and colleagues (42) who evaluated, based on the central role of heat shock protein 72 (HSP72) and TLR4 in innate response to bacterial infection, whether *HSPA1B A1267G* and *TLR4 A896G* genetic polymorphisms are risk factors for rUTI. They analyzed the prevalence of these two polymorphisms in 103 children with rUTI and 235 healthy controls. All of the children were assessed for VUR by VCUG and renal scarring by dimercaptosuccinic acid (DMSA) scan. *TLR4 A896G* genotype, *HSPA1B 1267G* and *TLR4 896G* alleles had significantly higher prevalence among rUTI patients than controls. Furthermore, *HSPA1B G1267G* genotype was associated with a higher risk of renal scarring even when adjusted for the presence of anatomical abnormalities including VUR. *TLR4 A896G* genotype and *896G* allele tended to occur more frequently in patients with rUTI without VUR than in those with vesicoureteral abnormalities. These data indicate that *HSPA1B 1267G* and *TLR4 896G* alleles, which have been associated with decreased *in vitro* signaling in response to lipopolysaccharide (LPS) as well as decreased *in vivo* bronchial airway responsiveness (43), are a risk factor for development of rUTI in childhood, independent of other renal abnormalities that predispose to the disease.

Yin et al. (44) confirmed increased frequency of *TLR4 896G* allele in 129 patients with UTI compared to 248 healthy controls and went further to examine TLR4 expression on peripheral blood monocytes. They found significantly lower TLR4 expression in patients with rUTI than in those with acute UTI or healthy controls, suggesting that *TLR4 896G* allele leads to UTI susceptibility by decreasing TLR4 expression and, consequently, bacterial clearance in urinary tract.

Tabel et al. (45) investigated distribution of a SNP within conserved part of the C-terminal region of human *TLR2 (Arg753Gln or G2258A)*, leading to a decreased activation of TLR2. The study was performed among 124 Turkish children with a history of a single UTI (*n* = 24) or rUTI (*n* = 100), and no VUR or other anomalies of urinary tract, and 116 unrelated adult healthy controls. They found that *TLR2 Arg753Gln* allele frequency was not just higher in the patient group when compared with control group but was also significantly higher in patients with rUTI than in those with a single UTI. Therefore, the authors suggested *TLR2 SNP G2258A* as a predisposing factor for rUTI.

Future studies are needed to confirm these genotypes as predisposing factors for rUTI in different populations, as well to explore the association of the investigated genotypes with TLR expression in patients with rUTI.

### Interferon Regulatory Factors 3 and 7

The activation of TLRs leads to induction of transcription factors like interferon regulatory factors (IRF) 3 and 7 (46). Recently, the involvement of IRFs in antibacterial defense and immunoregulation by TLRs has received more attention, since nuclear factor (NF)-κB, IRFs and activator protein (AP)-1 form transcriptional complexes that regulate innate immune responses in monocytes (47). In monocytic cells, IRF-3 and IRF-7 form heterodimers and the ratio plays an essential role for the inducible expression of type I interferon genes. Fischer and coauthors (48) showed that *IRF3* knockout mice develop severe acute pyelonephritis and extensive renal tissue damage in experimental UTI. Based on the phenotype of *IRF3* knockout mice, the authors predicted that reduced IRF3 expression could also increase human susceptibility to severe kidney infection. Therefore, IRF3 promoter sequence variation was studied in two patient populations with rUTI: 64 children (21 with rUTI, 16 with asymptomatic bacteriuria, 27 healthy controls) and 134 adults (72 with a childhood rUTI history and 62 healthy blood donors). DNA sequencing of *IRF3* promoters revealed significantly higher frequency of single nucleotide polymorphisms (SNPs) at -925 and -776 positions in rUTI patients than in those with asymptomatic bacteriuria (ABU) or healthy controls. Most of the rUTI patients (up to 80%) were homozygous for the two positions (*A/A–C/C*) compared to less than 20% of controls. These SNPs were associated with a lower IRF3 transcriptional activity.

On the other hand, Puthia and colleagues (49) found that *IRF-7* knockout mice experience lower bacterial burden in response to experimental UTI. Based on the protective phenotype of *IRF-7* knockout mice, the authors suggested that variant IRF7 expression might affect human UTI susceptibility. Indeed, *IRF7* promoter polymorphisms with lower IRF7 expression were found to be protective against recurrent acute pyelonephritis in children. Remarkably, all of the patients with rUTI (*n* = 51) were homozygous for the major allele rs3758650-C, which confers high IRF7 expression. By contrast, a proportion of the children with ABU were heterozygous for rs3758650-T, which confers lower IRF7 expression. An identical association was observed for the linked rs10902179 allele. Finally, the authors identified IRF-7 as a target for immunomodulatory therapy. Administering liposomal IRF-7 siRNA to IRF-3 knockout mice suppressed mucosal IRF-7 expression, and the mice were protected against infection and renal tissue damage. Therefore, IRF7 suppression was comparable to antibiotic therapy in regards to preventing renal abscess formation and damage.

With these findings in mind, IRF3 and IRF7 seem to exhibit opposing effects during UTI and balance each other in order to achieve an effective but limited innate immune response to bacterial infection. Further studies are needed to evaluate the role of these two transcription factors as predictors of developing rUTI or as novel therapeutic targets.

### Neutrophil Gelatinase-Associated Lipocalin

The activation of TLR4 by lipopolysaccharides induces neutrophil gelatinase-associated lipocalin (NGAL) expression in the alpha-intercalated renal cells and its secretion in urine (50). NGAL is an iron-transporting protein important for the clearance of bacteria in urinary tract through iron sequestration. Indeed, NGAL knock-out mice showed decreased clearance of bacteriuria following transurethral inoculation compared with controls (50).

Forster et al. (51) found uNGAL significantly decreased in children with UTI recurrence. The study involved 15 children (aged 6 months–21 years) with history of rUTI but without renal dysfunction or an active UTI and 15 healthy controls. Median urinary NGAL levels were lower in rUTI patients than in controls suggesting that defective local NGAL production predisposes to UTI recurrence. This might result from reduced TLR4 expression and reflect defective innate immunity. However, the study limitations include small number of patients and controls as well as the lack of sex and age matching between them. Because it has been reported that uNGAL levels increase with age (52), the lack of age matching might have lead to false positive results. Furthermore, 33% of the controls suffered from constipation which is frequently associated with UTI in children, though has not been shown as an independent causative factor in rUTI (24, 53). Finally, in this study uNGAL levels were not standardized with urine creatinine and the levels were compared only between children with rUTI and healthy children and not with children with a single UTI.

In a subsequent study Forster and colleagues (54) aimed to confirm the finding of decreased uNGAL levels in children with rUTI compared to those with a single UTI, but in this study only girls under the age of 4 were enrolled. Moreover, controls weren’t healthy children but girls who presented to the emergency department with signs and symptoms of a UTI but had a negative urine culture. First urine sample was taken at the time of acute UTI and follow-up urine sample was taken 2 weeks following the completion of the antibiotic course prescribed to treat the acute UTI. In this study, the authors have not found difference in uNGAL levels between children with single or recurrent UTI. However, this study also has a few limitations. Firstly, the follow-up urine samples were provided by only 53% of the initially enrolled children, including 3 controls, 24 children with single UTI and 10 children with rUTI, which limits the power of analysis of the follow-up uNGAL concentrations. Secondly, the follow-up urine sample was taken 2 weeks after antibiotic course which may have not been enough time after acute UTI for uNGAL to return to baseline levels, since the study on rats (55) showed increased uNGAL levels until 6-weeks time point after the bacterial injection. Additionally, control urine culture or urine analysis at the time of follow-up urine sampling was not reported, and therefore an ongoing subclinical inflammation was not excluded. Furthermore, in this study, opposite to prior, bowel or bladder dysfunction in children were not excluded. Finally, instead of healthy controls, children with signs and symptoms of UTI but with sterile urine culture were used as controls. Therefore, it is fair to assume these patients might have suffered from other unrecognized illneses that may influence the uNGAL levels (vasculitis, sterile pyuria, etc.).

In both of these studies (51, 54) children with former UTIs were included, which might have affected the study results, since renal scarring, which might occur as a consequence of previous UTIs and was not excluded in these patients, may also contribute to increased levels of uNGAL (55, 56). Indeed, a study by Lorenzo-Gomez et al. (57) reported increased uNGAL, transforming growth factor ß 1 (TGFß-1) and N-acetyl glucosaminidase (NAG) in elderly with rUTI history even in between acute UTIs. The study included 200 institutionalized elderly people (> 65 years) divided in rUTI and no rUTI group (100 subjects in each group) with higher serum creatinine and glomerular filtration rate in the rUTI group suggesting an already existing renal damage. Therefore, for using these markers as predictors of UTI recurrence initially present renal scarring should be excluded.

Yamanouchi and colleagues (58) also tested the hypothesis that reduced uNGAL predisposes children to rUTI in a prospective study. This study enrolled 38 children with first lifetime UTI who were subsequently divided in rUTI and single UTI groups according to recurrence of UTI over 3 years since the first UTI. The study included 20 age-matched healthy controls. They found that uNGAL levels (corrected by urinary creatinine, Cr) were significantly lower in the rUTI than in the single UTI group and age-matched healthy controls. The area under the receiver operating characteristic curve of NGAL/Cr was 0.86 for predicting recurrence of UTI. The group concluded that reduced uNGAL levels are a risk factor for rUTI and could serve as a biomarker. The strengths of this study include the evaluation of the following additional UTI risk factors in all patients: age, sex, presence and grade of VUR by VCUG and a presence of renal scars by DMSA scintigraphy. Interestingly, the prevalence of renal scarring did not differ between the recurrent and non-recurrent group, while the prevalence of high-grade VUR (grades III-V) was significantly higher in the recurrent group. Taken all together, this study confirmed that low levels of uNGAL at the non-infected stage could serve as an independent risk factor for rUTI.

However, it must be noted that in all these studies the measurement of uNGAL at the non-infected stage was performed only once for each patient. Moreover, none of these studies distinguished monomeric from dimeric uNGAL molecular form which might be of a great importance because the monomeric form is predominantly secreted by tubular epithelial cells, whereas the dimeric form is predominantly secreted by neutrophils (59). Therefore, future studies with differentiation of these two uNGAL forms and several measurements of uNGAL levels in each patient are necessary.

### Interleukin 8 and Its Receptors

Interleukin (IL) -8 (also known as CXCL8) transcription in urothelial cells is also induced by TLR4 activation. IL-8 has been shown to support neutrophil migration across the infected urothelium (60). IL-8 responses occur in patients with UTIs and show a correlation to urinary neutrophil numbers (61). IL-8 has two cell surface receptors on urothelial cells: CXC chemokine receptor 1 (CXCR1 or IL-8RA) and CXC chemokine receptor 2 (CXCR2 or IL-8RB) (62). Frendeus et al. (63) demonstrated that mice lacking the CXCR1 receptor are unable to clear uropathogenic bacteria and develop severe renal disease. Neutrophils were recruited to the site of infection but were unable to cross the epithelial barrier and were trapped in the tissues. The accumulation of neutrophils caused severe tissue destruction, and surviving mice developed renal scarring. Based on these findings, the authors investigated neutrophil CXCR1 expression in 12 children with rUTI but without an active infection (7 of whom had VUR) and in 12 age-matched controls. Neutrophils were obtained from the patients on two occasions with 1 year interval. Pairwise analysis showed consistently lower both cell surface CXCR1 expression (but not CXCR2) and CXCR1 mRNA levels in the patient neutrophils compared with age-matched controls. Additionally, by biweekly sampling of an individual with low levels of CXCR1 over a one year period the authors proved that the low CXCR1 expression is stable over time. Therefore, a reduced neutrophil CXCR1 expression seems to be a risk factor for developing rUTI, independent of VUR.

Lundstedt and coworkers validated these findings in two subsequent studies (64, 65). In the first study (64) the authors enrolled 8 children with rUTI history (6 of whom had VUR), 2 with a single UTI history and 49 of their adult relatives and compared the CXCR1 expression to 14 age matched children without UTI history and 46 adult female controls. They found a significantly decreased CXCR1 expression in patients and their relatives (regardless of UTI history or VUR presence) compared to controls. Authors suggested that low CXCR1 expression is inherited and is one of the factors predisposing to rUTI and therefore might be quantified when children present with their first episode of UTI to predict future recurrence. In their second research (65), the group examined *CXCR1* DNA sequences in two independent groups of individuals susceptible to UTI. The first group consisted of prospectively enrolled children (*n* = 24) who were followed from their first UTI episode and 26 age-matched controls. The second group enrolled 36 adult patients who had a history of rUTI, with a median of 30 years after the initial UTI episode. Adult healthy blood donors (*n* = 200) were included to assess the frequency of *CXCR1* sequence variants in the background population. Five sequence variants were detected in the intron of CXCR1: variant 1 (*217C/G*), variant 2 (+ *2608G/C*), variant 3 (+ *3081C/T*), variant 4 (+ *3082G/A*) and variant 5 (+ *3665G/A*). The results showed that these SNPs in *CXCR1* are associated with susceptibility to rUTI in both children and adults. Interestingly, rUTI patients without VUR had a higher frequency of *CXCR1* sequence variants than those with VUR, suggesting that *CXCR1* sequence variation and VUR are independent risk factors for developing rUTI. The group also associated these SNPs with reduced CXCR1 expression suggesting that *CXCR1* variants may render individuals UTI-prone by lowering CXCR1 expression and by incapacitating the neutrophil-dependent host defense against uropathogens.

On the other hand, Smithson et al. (66) found lower expression of neutrophil CXCR2 in 20 premenopausal women suffering from rUTI (without VUR) compared to 30 healthy female controls, suggesting that a low level of CXCR2 expression may increase the susceptibilities of premenopausal women to urinary tract infections. Low CXCR1 expression was detected in 3/9 patients with childhood APN, but the numbers were too small to draw any additional conclusions.

Javor et al. (67) evaluated the role of six selected functional polymorphisms in genes encoding IL-8 or its receptors in susceptibility to rUTI. The study enrolled 147 Slovak children divided in two subgroups: 85 with a single UTI and 62 with rUTI, and 215 unrelated healthy controls. Even though no differences were found in any of *IL-8* polymorphisms between patients and controls, subgroup analysis showed significantly higher frequency of -*251A* allele (a SNP in promoter that is associated with increased IL-8 production) in patients with rUTI compared to those with a single UTI. Furthermore, this study was the first to confirm the finding of Lundstedt et al. (65) by reporting a significant association between carrying *CXCR1* + *2608 C* allele and rUTI development independently of VUR, while carriers of the T allele of *CXCR2* + *1208 C/T* SNP have reduced risk of developing rUTI.

Recently, Han and colleagues (68) performed a meta-analysis of articles concerning *CXCR1* SNPs *rs2234671* and *rs3138060* published until the end of 2017. Their results showed no evidence of correlation between *CXCR1 rs2234671* polymorphism and susceptibility for UTI in adults, but found a significantly increased risk of UTI in children carrying *rs2234671*. This finding remained the same after excluding rUTI patients with VUR.

All of the above evidences indicate that the level and function of IL-8 and its receptors contribute to UTI susceptibility and therefore seem to be worth of exploring in larger, prospective studies of rUTI patients.

### Colony Stimulating Factors

Bacterial colonization and invasion of the urothelium triggers the production of granulocyte colony stimulating factor (G-CSF) which induces neutrophil emigration from the bone marrow (69). A study in mice with acute bacterial cystitis revealed significantly higher levels of serum G-CSF and IL-5 at the time of initial UTI in mice which later developed recurrent UTI than in those without UTI recurrence (70). Hannan et al. investigated in a subsequent study whether serum cytokines could identify patients susceptible to rUTI (71). The study involved 86 premenopausal women with acute uncomplicated cystitis (*Escherichia coli*) that were followed up for 3 months to determine UTI recurrence. In their initially taken serum samples 48 cytokines and growth factors were measured. During the 3 month follow-up, 41 women developed rUTI. Levels of IL-3, IL-8, CXCL1 and macrophage CSF (M-CSF) were elevated in patients who subsequently developed rUTI, but the expression level differed significantly only for M-CSF (amongst 48 different cytokines). Interestingly, the difference was most pronounced in the subset of patients who had first ever UTI at the time of study enrolment. This is in line with findings of the above mentioned study in mice (70), suggesting that higher levels of systemic inflammatory markers involved in myeloid cell inflammation (and consequently urothelial barrier damage) during the first ever episode of UTI is associated with UTI recurrence. Therefore, these findings indicate that high levels of neutrophil-mediated damage within the urothelial barrier contribute to host susceptibility to rUTI. Nevertheless, the limitations of this study include involvement of women with the history of rUTI as well as the short time of follow-up. Hence, it might be interesting to validate these findings in a prospective study of children presenting with a first time UTI and with a longer follow-up.

### Prostglandin E2

Studies performed in mouse models have implicated cyclooxygenase-2 (COX-2)–mediated inflammation as a key sensitizing factor for rUTI (70, 71). Hannan et al. (71) found that excessive urothelial neutrophil infiltration and COX-2–dependent inflammation cause tissue damage and remodeling that sensitize the bladder to severe rUTI. Furthermore, authors revealed that disruption of neutrophil response by inhibition of COX-2 early during UTI protects against UTI recurrence in mice and, therefore, suggested targeting COX-2 in the prevention and treatment of rUTI. These results were in accordance with their previous findings that dexamethasone (supressor of COX-2 inflammatory pathway) is protective against chronic and recurrent cystitis (70).

Based on these findings, Ebrahimzadeh and colleagues (72) assessed urinary prostaglandin E2 (PGE2), which is a product of arachidonic acid conversion by the COX-2 enzyme, as a biomarker for rUTI in a cohort of 92 postmenopausal women (31 women with acute UTI and a rUTI history, 35 women with rUTI but without an active infection and 26 healthy controls). They found a positive association between urinary PGE2 concentration and urothelial COX-2 expression. Furthermore, the authors divided patients into the above median PGE2 concentration group and below median group and recorded time to relapse over a 12-months period. They found that urinary PGE2 concentration in an acute UTI is predictive of rUTI relapse (3.5 times higher risk of UTI relapse in postmenopausal women with above median urinary PGE2 levels). However, during the remission stages, urinary PGE2 levels returned to normal concentrations suggesting the possible use of urinary PGE2 as a predictor of UTI recurrence only if measured during the acute stage of the initial UTI.

Prospective studies in larger cohorts are necessary to verify PGE2 as a predictor of UTI recurrence as well as to evaluate the role of COX-2 inhibitors in the prevention and treatment of rUTI.

### Nerve Growth Factor

Urinary nerve growth factor (uNGF) is a prototypical growth factor responsible for C-fiber afferent nerve excitability and reflex bladder activity (73). NGF is widely expressed in urothelial cells, bladder smooth muscle cells and mast cells where it serves as a mediator in the modulation of urothelial response to inflammation and altered pain signaling (74). In a prospective study Chuang and coworkers (75) evaluated whether uNGF levels could serve as a predictor of UTI recurrence in women following an acute UTI episode. Among the 37 included women (14 with a first time UTI and 23 with a recurrent UTI at the time of the enrollment), 9 women had UTI recurrence during 1 year follow-up. The study also enrolled 20 women with stress urinary incontinence as controls. uNGF levels were measured at baseline, weeks 1, 4 and 12. They found that the serial uNGF levels in women who developed UTI recurrence within 1 year were significantly lower than in women without recurrence, suggesting that lower uNGF levels could predict recurrent UTI. Although interesting, the study results are limited by the small cohort and use of women with stress urinary incontinence as controls, since uNGF levels are significantly increased in women with overactive bladder (76), so further studies are necessary to validate uNGF as a predictor of rUTI.

### Alpha Defensins

Human Neutrophil Peptides 1–3 (HNP1–3) are antimicrobial peptides that appertain to the alpha defensins family. HNP1–3 are encoded by the *DEFA1A3*, a multiallelic gene whose copy number polymorphisms (CNPs) may represent excellent candidates for disease risk modifiers because of their ability to generate significant gene dosage effects. Auxiliary studies to the Randomized Intervention for Children with Vesicoureteral Reflux (RIVUR) trial found that *DEFA1A3* is expressed in renal epithelium and not restricted to myeloid-derived cells and that genetic CNPs of the alpha defensin *DEFA1A3* locus in children with VUR can predict rUTI (77). The study included 298 patients (girls < 6 years) and 295 healthy controls (girls < 18 years). Patients in the RIVUR study had lower copy number of *DEFA1A3* compared to healthy controls; 29% of patients and 16% of controls had less than or equal to five copies of *DEFA1A3*. Moreover, for each additional copy of *DEFA1A3*, the odds of recurrent UTI in patients receiving antibiotic prophylaxis (so-called breakthrough infections) decreased by 47% when adjusting for VUR grade. In conclusion, low *DEFA1A3* copy number was found to be a risk factor for a breakthrough UTI independent of VUR grade in subjects in the RIVUR Study. The authors found a significant correlation between *DEFA1A3* copy number and kidney mRNA expression. However, further studies should evaluate whether urinary HNP1-3 levels correlate with *DEFA1A3* copy number and whether urinary HNP1–3 levels could be used for predicting rUTI.

### Tamm-Horsfall Protein

Tamm-Horsfall protein (THP) is an evolutionary conserved glycoprotein synthesized only in kidneys and is one of the most abundant urinary proteins (78). THP serves as an effective soluble receptor for type 1-fimbriated *E. coli*, competitively inhibiting their adherence to the uroplakin Ia receptors on the urothelial surface and therefore prevents colonization of the urinary tract (78). Studies on mice revealed increased UTI susceptibility in THP knockout mice (79, 80). A few research groups have investigated a potential use of urinary THP levels for recognizing patients with a greater risk of developing rUTI (81–83). Lose et al. (81) were the first to compare urinary THP levels between 16 girls aged 5–17 years with rUTI history, but without VUR or renal scarring, and 14 age-matched healthy girls and found no significant differences, while Reinhart et al. validated these findings in a longitudinal study including 17 young women (mean age 24) and 18 controls (82) and later confirmed the same results in children (girls, mean age 7.9) (83). Taken all together, since no significant differences have been found between patients with rUTI and controls, the level of urinary THP does not seem to have a role in predicting UTI recurrence.

### Surfactant Proteins

Surfactant protein (SP) -A has been recently recognized as an immunomodulator in urinary tract infections (84). SP-A facilitates pathogen clearance by enhancing opsonic and non-opsonic phagocytosis *via* binding to SP-A receptor in macrophages (84). Hu et al. (85) showed that knockout of SP-A and SP-D in a murine model of uropathogenic *E. coli-*induced UTI increases bacterial loads and neutrophil infiltration in the kidneys, indicating that SP-A and SP-D may attenuate kidney infection by inhibiting bacterial growth and modulating renal inflammation. Liu et al. (86) investigated whether polymorphisms in SP- A (*SP-A1* and *SP-A2*) and D genes (*SP-D*) are associated with the risk of rUTI. The study enrolled 32 female patients with history of rUTI and 30 age-matched unrelated female healthy controls. The frequencies of *19Ala* allele of *SP-A1* and *223Gln* allele of *SP-A2* were significantly higher in patients than in controls. Furthermore, serum SP-A and SP-D levels were increased whereas urinary SP-A and SP-D levels were decreased in patients compared to controls. The *19Ala/Ala* and *223Gln/Gln* genotypes in rUTI patients were associated with high serum and low urine SP levels. The authors speculated that *19Ala* allele of *SP-A1* and *223Gln* allele of *SP-A2* gene lead to increased rUTI susceptibility due to low urinary SP-A expression and therefore weaker capacities to regulate host innate immunity. Nevertheless, future studies are necessary to validate urinary SP-A levels as biomarkers for predicting rUTI.

### Immunoglobulins

Suman et al. (87) reported that serum levels of immunoglobulin (Ig) G, IgA and IgM are significantly higher in women with rUTI after completing antibiotic therapy than in healthy controls. The difference was also significant for urinary IgG. In addition to serum immunoglobulins, secretory IgA (sIgA), synthesized locally in the uroepithelium, is also excreted in urine (88). An *in vitro* study (89) demonstrated that sIgA prevents bacterial adhesion to the uroepithelium. Riedasch et al. (90) and Fliedner et al. (91) investigated urinary secretory IgA (sIgA) in women with rUTI. The first study (90) showed low urinary sIgA concentrations in women with rUTI, even in an acute UTI. However, women with an acute UTI without a history of UTI also had low urinary sIgA. Another study (91) compared urinary sIgA levels in children with rUTI to those of children with an acute UTI. Children with history of rUTI but without an acute UTI episode or urinary tract abnormalities had significantly lower sIgA than healthy controls. Furthermore, urinary slgA excretion rate was higher in children with acute UTI than controls, with the highest values in those with abnormal urinary tracts. Therefore, it might be worth to compare urinary sIgA levels in same patients with rUTI history in both acute UTI and inbetween acute UTIs in future studies. However, although sIgA might serve as predictor of rUTI, it is impractical to use in everyday clinical practice since consistent but variable losses of sIgA occur when urine is concentrated or stored, so immediate determination of sIgA at the time of urine sampling should be done. In addition, serum IgA in urine may interfere with sIgA measurements. Furthermore, urinary sIgA concentrations change with age and with the menstrual cycle in women, which the two before mentioned studies did not take in consideration (92). Finally, additional studies with exclusion of the serum IgA interference in urine samples and age or menstrual cycle phase influence (93) found no association of UTI recurrence with disturbances of the urinary sIgA excretion, including even a complete failure of the sIgA system. In conclusion, the local deficit of sIgA seems not be useful as a rUTI predictor.

### Vitamin D

Vitamin D has a crucial role in enhancing innate immunity, mainly by increasing the neutrophilic motility, phagocytic function and the expression of potent antimicrobial peptides including cathelicidin (94, 95). Uroepithelium-derived cathelicidin (also known as human LL-37) protects the urinary tract from bacterial adherence by preventing biofilm formation and a direct antimicrobial activity by disrupting bacterial membranes, leading to cell lysis (96). Nielsen et al. (97) performed study on 50 patients with UTI and 53 healthy controls and observed a significant association between urine cathelicidin level and incidence of UTI. The authors found that an acute UTI causes an increase in urinary cathelicidin levels. Additionally, they observed significantly lower cathelicidin production in subjects susceptible to UTI, but without an acute UTI, than in a comparable control group that never had an UTI, indicating that reduced cathelicidin production in the urinary tract system raises the probability of developing UTI.

Vitamin D induces cathelicidin production in uroepithelial cells by binding to the vitamin D responsive element (VDRE) in the cathelicidin gene promoter (98). Hertting et al. (99) observed that vitamin D supplementation leads to a significant increase in cathelicidin production in biopsy samples of urinary bladders infected with uropathogenic *E. coli*. Therefore, the authors suggested that vitamin D supplementation prevents UTI by increasing urine cathelicidin concentration. Övünç Hacıhamdioğlu et al. (100) reported that children with vitamin D deficiency are not able to increase their urine cathelicidin level during UTI, in contrast to those with sufficient vitamin D levels, indicating that sufficient concentrations of circulating vitamin D are mandatory for optimal cathelicidin production. Therefore, it seems that vitamin D deficiency results in failure to produce adequate antibacterial peptides and, consequently, predisposes the individual to UTI. However, the study sample size was low (36 children with UTI and 38 healthy controls) and the study design was cross-sectional, demanding prospective studies to validate the beneficial effect of vitamin D supplementation to restore cathelicidin expression and prevent UTI recurrence.

The study performed by Tekin et al. (101) on 82 children (aged 2–18 years) with UTI, and no other risk factors for UTI, confirmed vitamin D deficiency as an independent risk factor for UTI in children. Children with low serum vitamin D levels (< 20 nmol/l) were 3.5 times more likely to develop UTI than those with normal levels. The reported results were later verified in multiple studies (102–104). Yang et al. (105) found, in addition to significantly lower serum vitamin D level in infants with UTI than in healthy infants, that vitamin D supplementation is associated with a decreased likelihood of UTI. Moreover, significantly lower serum vitamin D levels in infants with pyelonephritis than in infants with lower UTI were observed. Finally, a meta-analysis conducted on nine studies investigating serum vitamin D levels in patients with UTI confirmed a direct link between vitamin D insufficiency and an increased risk of UTI (106). However, all of these studies compared patients with first time acute UTI with healthy controls, and none of the mentioned studies involved patients with rUTI.

Nseir et al. (107) reported significantly lower mean serum levels of vitamin D among premenopausal women with rUTI compared with age-matched healthy controls. Vitamin D deficiency in this retrospective study was independently associated with recurrent UTIs, though a relatively small number of subjects was included (93 patients with a history of rUTI). Georgieva et al. (108) validated vitamin D deficiency as a risk factor for UTI and examined further the association between serum vitamin D level and UTI recurrence by prospectively following UTI patients for 1 year. Out of 76 children with UTI, 21 (28%) had a recurrence within one year after the index infection, but the authors did not find an association between serum levels of vitamin D, cathelicidin and recurrence of UTI. This may be due to a small number of patients included and inclusion of patients with other risk factors for UTI such as VUR and other congenital urinary tract abnormalities. More recently, Muntean and Sasaran (109) reported incontestably lower values of serum vitamin D levels in patients with recurrent UTI than in the ones with a first-time UTI but without recurrence. Still, this study also included a relatively small number of patients as its major limitation. Therefore, future follow-up study to evaluate the incidence of UTI in patients with rUTI and prior vitamin D deficiency after reaching normal serum values of serum vitamin D would be of a great interest.

Furthermore, genetic susceptibility to UTI and renal scarring has been linked to vitamin D receptor (*VDR*) gene polymorphisms (110, 111). *VDR* alleles may cause alterations in VDR function, which can affect resistance or susceptibility to infections. The study of Aslan et al. on 92 children with UTI (case group) and 105 healthy children (control group) showed that *VDR* gene *Fok1 ff* genotype is associated with 3.94 times greater risk of UTI and renal scar formation than *FF* genotype, while *Apa1 Aa* or *aa* genotype seem to be protective factors (110). On the other hand, Mahyar et al. reported a significant difference between the case (*n* = 60) and the control (*n* = 60) groups for *VDR* gene *Apa1* and *Bsm1* polymorphisms, but not for *Fok1* and *Taq1* polymorphisms (111). In this study *Apa1* a allele and *Bsm1* b allele were associated with increased risk of UTI. Association between *VDR* polymorphisms and UTI susceptibility is in accordance with the crucial role that vitamin D plays in modulation of the immune response against uropathogens, so further studies in larger cohorts with simultaneous measurement of serum vitamin D and *VDR* gene polymorphisms are required.

Based on all of these findings, vitamin D supplementation for prevention of rUTI has become a topic of interest in many recent studies, especially since several interventions, other than antibiotic prophylaxis, for the prevention of recurrent UTI have been tried but so far did not provide a definitive effective answer (112). Jorde et al. (113) conducted a randomized controlled trial on patients with prediabetes who were randomized to vitamin D3 (20,000 IU per week) versus placebo for five years (116 subjects who received vitamin D and 111 who received placebo completed the five-year study). During the 5 year follow up, 18 subjects in the vitamin D group and 34 subjects in the placebo group developed UTI. Therefore, supplementation with vitamin D significantly reduced the occurrence and number of UTI during this five years long intervention study. However, questionnaires with self-reported occurrence of UTI without any bacteriological verification were used in the study. It is also remarkable that the protective effect of vitamin D was significant not only in all subjects analyzed together, but also in those with sufficient baseline serum vitamin D level (above 50 nmol/L). These results are in contrast to those obtained by Merrikhi et al. (114) who performed a randomized, triple-masked control trial among children with rUTI. Administration of oral vitamin D drops with a dose of 1000 IU/daily did not prove to bring a significant benefit in preventing recurrence of UTI in this study. However, the study is limited by a small number of subjects included (68 children) and a short follow-up time (6 months). It is possible that the protective effect of vitamin D on rUTI would be more emphasized when reaching normal or higher serum level of vitamin D by using higher dose of supplementation (> 1000 IU/daily) or by prolonged duration of its administration (> 6 months). Therefore, interventional studies evaluating the role of vitamin D supplementation to reduce the burden of rUTI with a greater number of subjects and longer follow-up are warranted.

## Conclusion

Recently discovered essential role of urothelial innate immunity mechanisms in defense against uropathogens has led to numerous research investigating innate immunity components as biomarkers of UTI recurrence. The interindividual variability in susceptibility for rUTI seems to be caused by genetic polymorphisms which alter expression of innate immunity components and therefore influence efficacy of host response to invading uropathogens. Though multiple genetic polymorphisms and certain immunity components deficiencies have been suggested as risk factors for developing rUTI, replication studies in larger cohorts of different populations are necessary to confirm the observed genotype-phenotype associations and biomarkers predictive of rUTI.

**FIGURE 2:**
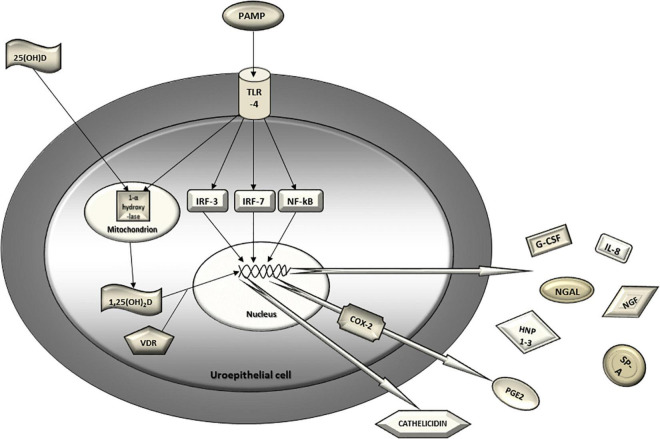
Urothelial innate immunity mechanisms in defense against uropathogens. Uroepithelial cell detects uropathogen *via* pattern recognition receptors (TLR4). The triggered intracellular signaling cascade results in the activation of nuclear transcription factors (IRF3, IRF7, NF-kB) and ultimately leads to increased gene transcription, production and secretion of chemokines, cytokines and antimicrobial peptides into the urinary stream. Activation of TLR4 also leads to induction of COX-2 expression in the uroepithelial cell and, consequently, increased urinary PGE2. 25-hydroxy vitamin D from circulation converses to 1,25-dihydroxy vitamin D in urothelial cell by mitochondrial 1-α hydroxylase which is also induced by activated TLR4. Finally, 1,25-dihydroxy vitamin D binds to the vitamin D receptor in cytoplasm and translocates to the nucleus where it binds to the vitamin D responsive element and induces transcription of a potent antimicrobial peptide called cathelicidin. PAMP, pathogen associated molecular pattern; TLR, Toll-like receptor; IRF, interferon regulatory factor; NF-kB, nuclear factor kappa B; 25(OH)D, 25-hydroxy vitamin D; 1,25(OH)_2_D, 1,25-dihydroxy vitamin D; VDR, vitamin D receptor; IL-8, interleukin 8; NGAL, neutrophil gelatinase associated lipocalin; NGF, nerve growth factor; G-CSF, granulocyte colony stimulating factor; SP-A, surfactant protein A; HNP1-3, human neutrophil peptides 1-3; COX-2, cyclooxigenase 2; PGE2, prostaglandin E2.

Uroepithelial cells detect uropathogens *via* pattern recognition receptors (mainly TLR4) which triggers intracellular signaling cascade resulting in the activation of nuclear transcription factors (IRF3, IRF7, NFk-B) and ultimately leads to an increased production and secretion of chemokines, cytokines and antimicrobial peptides into the urinary stream (Figure 2). In the light of recent evidence, TLR4, transcription factors IRF 3 and 7, IL-8 and its receptors, PGE2, serum vitamin D and urinary antimicrobial peptides (NGAL, cathelicidin, HNP1-3 and SP-A) have been discerned as promising predictors of UTI recurrence. Current armamentarium suggests that a lack of a single component of the urinary tract innate immunity system might lead to an increased susceptibility for rUTI and therefore represent a clinically useful biomarker for predicting rUTI. Clinical studies of these urothelial cell receptors, nuclear transcription factors, cytokines, antimicrobial peptides and their genetic polymorphisms are opening a whole new area of research possibilities and, hopefully, era of significant advances in understanding and managing rUTI.

However, general conclusions from most of the published studies are limited by a small number of subjects included, cross-sectional and retrospective study design and the marked heterogenicity between studies. Moreover, none of the articles included in this systematic review provides evidence for a prophylaxis of rUTI after designating specific deficits of the urothelial innate immunity mechanisms in UTI-prone individuals. Among all of the proposed biomarkers for predicting UTI recurrence, only vitamin D supplementation has been investigated as rUTI prophylaxis in a few recent studies, but so far, due to conflicting results, with a debatable effectiveness. Therefore, the proposed biomarkers may represent the genetic, anatomical and functional deficits, but, for now, cannot provide the way of prophylaxis. Nevertheless, better understanding of the host innate immunity deficiency biomarkers associated with rUTI could help researchers tailor future studies of prophylactic strategies to effectively reduce the potential for UTI recurrence. In addition, most of the research includes mainly or exclusively female patients. This is probably due to a higher incidence of UTI in women, as well as a higher prevalence of urinary tract anatomical abnormalities in man. Interestingly, even in studies which had no gender limitations in selection of patients, after excluding those with anatomical abnormalities, most of the included patients were female. While possible solution for this and other biases might be a larger sample size, further prospective longitudinal studies of the antimicrobial activity, regulation, signaling and genetic variations of urinary immune system components are certainly warranted for better understanding of aberrant defense mechanisms that predispose individuals to rUTI, as well as for validation of the predictive efficacy of the proposed rUTI susceptibility biomarkers (Table 3).

**TABLE 3:** Proposed biomarkers for predicting urinary tract infections (UTI) recurrence and goals for future research.

Proposed biomarker for predicting UTI recurrence	Future research goals
Toll-like receptors (TLRs)	Future studies should validate *TLR* gene polymorphisms as predisposing factors for rUTI in different populations and explore the association of the investigated genotypes with TLR expression in patients with rUTI.
Interferon regulatory factors (IRF) 3 and 7	Further studies should confirm *IRF3* and *IRF7* genetic polymorphisms as risk factors for UTI recurrence in different populations. Additional studies should evaluate benefits of suppressing IRF7 expression by administering liposomal IRF7 siRNA on UTI recurrence.
Neutrophil gelatinase-associated lipocalin (NGAL)	Prospective longitudinal studies with differentiation of monomeric and dimeric urinary NGAL forms and several measurements of uNGAL levels in each patient after the first lifetime UTI are necessary to validate a specific NGAL form as a predictor of rUTI.
IL-8 and its receptors (CXCR1 and CXCR2)	Future studies should verify *CXCR1* genetic polymorphisms as risk factors for rUTI in different populations. Prospective longitudinal studies following patients from their first UTI episode should validate the role of CXCR1 neutrophil expression and IL-8 levels for predicting UTI recurrence.
Colony stimulating factors (CSF)	Prospective longitudinal studies should confirm the value of serum M-CSF and G-CSF levels as predictors of future rUTI in patients with a first time UTI.
Prostaglandin E2 (PGE2)	Prospective studies in large cohorts are necessary to verify urinary PGE2 concentration in acute first time UTI as a predictor of future UTI recurrence. Randomized controlled trials should evaluate the role of COX-2 inhibitors in the prevention and treatment of rUTI.
Nerve growth factor (NGF)	Prospective longitudinal studies in large cohorts are necessary to validate urinary NGF level after the first lifetime UTI as a predictor of UTI recurrence.
Human neutrophil peptides 1-3 (HNP 1-3)	Future studies should verify low *DEFA1A3* copy number polymorphisms as an independent risk factor for rUTI and evaluate whether urinary HNP1-3 levels correlate with *DEFA1A3* copy number. Prospective longitudinal studies should explore urinary HNP1-3 level as a predictor of rUTI.
Surfactant protein A (SP-A)	Prospective longitudinal studies in large cohorts are necessary to validate urinary and serum SP-A levels as biomarkers for predicting rUTI.
Vitamin D and cathelicidin	Prospective, longitudinal studies in large cohorts are warranted for confirming serum vitamin D and/or urinary cathelicidin level as an independent predictor of rUTI development after the first lifetime UTI. Future randomized controlled trials should verify the beneficial effect of vitamin D supplementation on urinary cathelicidin levels and prevention of UTI recurrence.

Hopefully, this review will inspire future attempts to further explore known and identify unknown biomarkers for recognizing rUTI susceptible patients who might benefit from more intense diagnostic surveillance and therapeutic intervention. Beside clear advantages for patients and healthcare through reduced morbidity, the use of antibiotics and/or invasive procedures, such biomarker(s) could lead to the development of new treatment options that facilitate bacterial clearance by modulating the host immune response.

## Data Availability Statement

The original contributions presented in this study are included in the article/supplementary material, further inquiries can be directed to the corresponding author.

## Author Contributions

IS wrote the manuscript. LL edited, supervised, and oversaw the manuscript. AC reviewed the manuscript and contributed to the final draft. All authors discussed the contents, contributed to the article, and approved the submitted version.

## Conflict of Interest

The authors declare that the research was conducted in the absence of any commercial or financial relationships that could be construed as a potential conflict of interest.

## Publisher’s Note

All claims expressed in this article are solely those of the authors and do not necessarily represent those of their affiliated organizations, or those of the publisher, the editors and the reviewers. Any product that may be evaluated in this article, or claim that may be made by its manufacturer, is not guaranteed or endorsed by the publisher.
